# rAAV-Mediated Overexpression of SOX9 and TGF-β via Carbon Dot-Guided Vector Delivery Enhances the Biological Activities in Human Bone Marrow-Derived Mesenchymal Stromal Cells

**DOI:** 10.3390/nano10050855

**Published:** 2020-04-28

**Authors:** Weikun Meng, Ana Rey-Rico, Mickaël Claudel, Gertrud Schmitt, Susanne Speicher-Mentges, Françoise Pons, Luc Lebeau, Jagadeesh K. Venkatesan, Magali Cucchiarini

**Affiliations:** 1 Center of Experimental Orthopaedics, Saarland University Medical Center, D-66421 Homburg, Germany; 2 Cell Therapy and Regenerative Medicine Unit, Centro de Investigacións Científicas Avanzadas (CICA), Universidade da Coruña, ES-15071 A Coruña, Spain; 3 Laboratoire de Conception et Application de Molécules Bioactives, Faculty of Pharmacy, UMR 7199 CNRS—University of Strasbourg, F-67401 Illkirch, France

**Keywords:** bone marrow-derived mesenchymal stromal cells, rAAV vectors, carbon dots, SOX9, TGF-β, cartilage repair

## Abstract

Scaffold-assisted gene therapy is a highly promising tool to treat articular cartilage lesions upon direct delivery of chondrogenic candidate sequences. The goal of this study was to examine the feasibility and benefits of providing highly chondroreparative agents, the cartilage-specific sex-determining region Y-type high-mobility group 9 (SOX9) transcription factor or the transforming growth factor beta (TGF-β), to human bone marrow-derived mesenchymal stromal cells (hMSCs) via clinically adapted, independent recombinant adeno-associated virus (rAAV) vectors formulated with carbon dots (CDs), a novel class of carbon-dominated nanomaterials. Effective complexation and release of a reporter rAAV-*lacZ* vector was achieved using four different CDs elaborated from 1-citric acid and pentaethylenehexamine (CD-1); 2-citric acid, poly(ethylene glycol) monomethyl ether (MW 550 Da), and *N*,*N*-dimethylethylenediamine (CD-2); 3-citric acid, branched poly(ethylenimine) (MW 600 Da), and poly(ethylene glycol) monomethyl ether (MW 2 kDa) (CD-3); and 4-citric acid and branched poly(ethylenimine) (MW 600 Da) (CD-4), allowing for the genetic modification of hMSCs. Among the nanoparticles, CD-2 showed an optimal ability for rAAV delivery (up to 2.2-fold increase in *lacZ* expression relative to free vector treatment with 100% cell viability for at least 10 days, the longest time point examined). Administration of therapeutic (SOX9, TGF-β) rAAV vectors in hMSCs via CD-2 led to the effective overexpression of each independent transgene, promoting enhanced cell proliferation (TGF-β) and cartilage matrix deposition (glycosaminoglycans, type-II collagen) for at least 21 days relative to control treatments (CD-2 lacking rAAV or associated to rAAV-*lacZ*), while advantageously restricting undesirable type-I and -X collagen deposition. These results reveal the potential of CD-guided rAAV gene administration in hMSCs as safe, non-invasive systems for translational strategies to enhance cartilage repair.

## 1. Introduction

Articular cartilage lesions represent serious clinical issues in orthopaedics as this specialized tissue does not fully heal on itself by lack of vascularization and of local chondroregenerative cells that may repopulate the defects [1,2]. Despite the availability of a number of clinical interventions (Pridie drilling, microfracture, cell transplantation), none can promote the generation of the original hyaline cartilage (proteoglycans, type-II collagen) in the lesions, with instead the appearance of a fibrocartilaginous repair tissue (type-I collagen) showing lesser mechanical properties and that may be prone to osteoarthritis [1,2,3,4]. Administration of chondroreparative mesenchymal stromal cells (MSCs) [5,6,7] in focal cartilage defects represents a valuable therapeutic alternative to activate the local healing processes [8,9], yet here again formation of the native hyaline cartilage is not observed [8,9], showing the necessity to develop improved treatments for adapted cartilage repair.

Scaffold-assisted gene transfer is an attractive therapeutic approach for cartilage repair as it has the potential to activate the intrinsic repair processes in sites of cartilage lesions by controlling the delivery of carriers coding for candidate genes [10,11,12], having been reported using nonviral [13,14,15,16,17,18,19] and lentiviral vectors [20,21,22,23]. While such gene vectors commonly support short-term transgene expression (nonviral vectors) or have the potential to activate oncogenes following genome integration (lentiviral vectors), vectors based on adeno-associated viruses (AAV) may be more adapted as they promote transgene expression over extended periods of time (some years) in a much safer manner due to the lack of viral protein coding sequences in the recombinant AAV (rAAV) backbone [10,11]. Thus far, biomaterial-assisted rAAV gene transfer for cartilage research has been described including polymeric micelles [24,25,26,27], hydrogels [28,29,30,31,32] and solid scaffolds [33], yet other materials may constitute valuable systems for rAAV delivery in experimental cartilage therapy. In this regard, carbon dots (CDs), a recently discovered class of carbon-dominated, biocompatible nanomaterials [34,35] used in drug delivery and theranostic approaches [35,36], may be good candidates to achieve this goal as they have been reported for their ability to intracellularly deliver nucleic acids and proteins in vitro [37] and in experimental models in vivo of cancer [38,39,40] and for regenerative medicine [41,42]. It remains to be seen whether CDs are capable of assisting rAAV vector transfer for cartilage repair as such vectors are more effective than nonviral vehicles to deliver genetic material in target cells [10,11].

The goal of this study was therefore to evaluate the potential of various CDs to associate with and release rAAV vectors as a means to target chondrogenically competent human MSCs (hMSCs), with a focus on transferring DNA sequences for the highly chondroreparative sex-determining region Y-type high mobility group box 9 (SOX9) transcription factor [43] and transforming growth factor beta (TGF-β) [5,6]. The data show that CDs are potent systems to efficiently vectorize and release rAAV, especially CD-2 nanoparticles, which allow hMSCs to be optimally targeted via rAAV gene transfer. Specific delivery of rAAV vectors carrying either the candidate SOX9 or TGF-β sequences assisted by CD-2 led to effective expression of the transgenes in these cells, enhancing cell proliferation and cartilage matrix deposition (glycosaminoglycans, type-II collagen) with reduced type-I and -X collagen production. These findings provide evidence on the ability of CD-assisted therapeutic rAAV gene delivery to target chondroreparative hMSCs in future non-invasive and safe applications to treat sites of cartilage injury.

## 2. Materials and Methods

### 2.1. Reagents

All reagents were purchased at Sigma (Munich, Germany) unless otherwise indicated. The anti-SOX9 (C-20) and anti-TGF-β (V) antibodies were from Santa Cruz Biotechnology (Heidelberg, Germany), the anti-type-II collagen (II-II6B3) from the NIH Hybridome Bank (University of Iowa, Ames, IA, USA), the anti-type-I collagen (AF-5610) antibody from Acris (Hiddenhausen, Germany), and the anti-type-X collagen (COL-10) antibody from Sigma. The biotinylated secondary antibodies and ABC reagent were from Vector Laboratories (Alexis Deutschland GmbH, Grünberg, Germany). The AAVanced Concentration Reagent was from System Bioscience (Heidelberg, Germany) and the Cy3 Ab Labeling Kit from Amersham/GE Healthcare (Munich, Germany). The AAV titration ELISA was from Progen (Heidelberg, Germany). The β-gal staining kit and the Cell Proliferation Reagent WST-1 were purchased at Roche Applied Science (Mannheim, Germany), the Beta-Glo^®^ Assay System at Promega (Mannheim, Germany), and the TGF-β Quantikine ELISA at R&D Systems (Wiesbaden, Germany).

### 2.2. Human Bone Marrow-Derived Mesenchymal Stromal Cells

The study was approved by the Ethics Committee of the Saarland Physicians Council (*Ärztekammer des Saarlandes*, reference number Ha06/08). All patients provided informed consent before being included in the study, which was performed in accordance with the Helsinki Declaration. Bone marrow aspirates (~15 mL; 0.4–1.2 × 10^9^ cells/mL) were prepared from the distal femurs of patients undergoing total knee arthroplasty (n = 12, age 75 ± 3 years). Bone marrow-derived human mesenchymal stromal cells (hMSCs) were isolated by washing and centrifuging the aspirates in Dulbecco’s modified Eagle’s medium (DMEM) and resuspending the pellet in red blood cell lysing buffer with DMEM (1:1) [44,45]. The mixtures were washed and resuspended in DMEM, 10% fetal bovine serum, 100 U/mL penicillin, and 100 μL/mL streptomycin (growth medium) for cell plating and maintenance in T75 flasks at 37 °C under 5% CO_2_. A medium change was performed after 24 h using growth medium with recombinant FGF-2 (1 ng/mL) for expansion [44,45], followed by changes every 2–3 days and replating when the cells reached a density of 85%, using cells at no more than passage 1–2.

### 2.3. Preparation of the Carbon Dots

**Figure 1:**
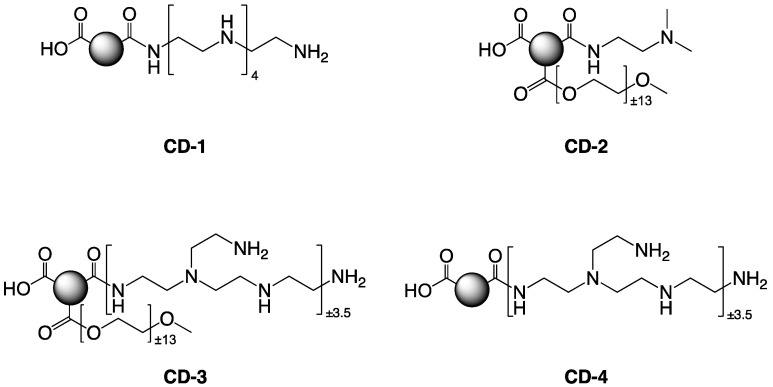
Structural features of the various carbon dots employed in the study. The nanoparticles CD-1 to CD-4 were generated through pyrolysis of citric acid (CA) in the presence of various passivation reagents presented in Materials and Methods and in Table 1.

The various carbon dots (CD-1 to CD-4) were generated through a bottom-up approach, using pyrolysis of citric acid (CA) as the carbon source, in the presence of various additives as passivation reagent: pentaethylenehexamine (PEHA), *N*,*N*-dimethylethylenediamine (DMEDA), branched poly(ethyleneimine) 600 Da (bPEI_600_), poly(ethylene glycol) monomethyl ether 550 Da (mPEG_550_), or 2 kDa (mPEG_2000_) [35,38] (Figure 1 and Table 1). Pyrolysis was conducted under conventional heating or microwave irradiation, and the resulting nanoparticles were purified using extensive dialysis against HCl 0.1 N and ultrapure H_2_O (MWCO 1000 Da) [35,38]. The CDs were freeze-dried, and 5.0 mg/mL stock solutions were prepared and stored at 4 °C until use [35,38]. The size and charge (zeta potential, ζ) of the nanoparticles were determined using dynamic light scattering (DLS) (NanoSizer NanoZS, Malvern UK) and transmission electron microscopy (TEM) operating at 5 kV (LVEM5, Delong Instruments, Brno, Czech Republic) [35,38] (Table 1).

### 2.4. Preparation of the rAAV Vectors

The vectors were generated using pSSV9, a parental AAV-2 genomic clone [46,47]. rAAV-*lacZ* carries the *E. coli* β-galactosidase (*lacZ*) reporter gene, rAAV-FLAG-h*sox9* a 1.7-kb FLAG-tagged human *sox9* (h*sox9*) cDNA sequence, and rAAV-hTGF-β a 1.2-kb human transforming growth factor beta 1 (hTGF-β) sequence, all controlled by the cytomegalovirus immediate-early (CMV-IE) promoter [25,44,45]. Conventional packaging of not self-complementary vectors was performed using helper-free (two-plasmid) transfection in 293 cells with the packaging plasmid pXX2 and adenovirus helper plasmid pXX6 [25,45]. Vector purification was performed using the AAVanced Concentration Reagent [25], and vector titers were monitored using real-time PCR [25,44,45], averaging 10^10^ transgene copies/mL (~1/500 functional recombinant viral particles).

### 2.5. Cy3 Labeling

The rAAV vectors were labeled using a Cy3 Ab Labeling Kit according to the manufacturer’s recommendations by mixing rAAV (1 mL) in sodium carbonate/sodium bicarbonate buffer (pH 9.3) for 30 min at room temperature, followed by labeling with Cy3 and dialysis against 20 mM HEPES (pH 7.5)/150 mL NaCl [25].

### 2.6. Complexation of the rAAV Vectors with the Carbon Dots and Release Studies

The various CDs (40 μL) were directly mixed with the rAAV vectors (40 μL, 8 × 10^5^ transgene copies) and incubated for 30 min at room temperature to generate the rAAV/CD systems. Alternatively, Cy3-labeled rAAV vectors were employed for the visualization analyses of the complexation studies by mixing Cy3-labeled rAAV (40 μL, 8 × 10^5^ transgene copies) with the CDs (40 μL) in 96-well plates in serum-free DMEM (100 μL). Cy3 labeling of the samples was monitored under live fluorescence with a rhodamine filter set (Olympus CKX41, Hamburg, Germany). For the release studies, the rAAV/CD systems as prepared above were placed in 24-well plates in 350 μL of serum-free DMEM, and rAAV was measured in aliquots of culture medium at the denoted time points using an AAV titration ELISA [25].

### 2.7. rAAV/CD-Mediated Gene Transfer

Monolayer cultures of hMSCs were directly incubated with the rAAV/CD systems prepared as described above in the various assays at the indicated cell densities, culture formats, and volume/multiplicity of infection (MOI) [25,44,45]. The cultures were maintained in growth medium [44,45] in a humidified atmosphere with 5% CO_2_ and at 37 °C for up to 21 days for the analyses.

### 2.8. Transgene Expression

*lacZ* expression was assessed using X-Gal staining for monitoring under light microscopy (Olympus BX45) and using the Beta-Glo^®^ Assay System to provide an estimation of the β-gal activity (values expressed as relative luminescence units—RLU—with normalization to the number of cells) [25]. Expression of SOX9 and TGF-β was monitored using immunohistochemical analysis with specific primary antibodies, a biotinylated secondary antibody, and using the ABC method with diaminobenzidine (DAB) as a chromogen for monitoring under light microscopy (Olympus BX45) [44,45]. TGF-β expression was also measured using specific ELISA [45]. All measurements were performed using a GENios spectrophotometer/fluorometer (Tecan, Crailsheim, Germany).

### 2.9. Cell Viability and Proliferation

Cell viability was monitored using the Cell Proliferation Reagent WST-1, with OD^450 nm^ being proportional to the number of cells [25,44,45]. Cell proliferation was provided as a direct index [44,45]. Cell viability percentage [25] was calculated as:

Cell viability (%) = (absorbance of the sample/absorbance of the negative control) × 100

All measurements were performed using a GENios spectrophotometer/fluorometer (Tecan).

### 2.10. Histology and Immunohistochemistry

Cells in monolayer cultures were harvested at the denoted time points for fixation in 4% formalin. Fixed cells were stained with alcian blue for glycosaminoglycans as previously reported [25], with removal of excess stain in double distilled water. The stain was quantitatively estimated using solubilization in 6 M guanidine hydrochloride overnight to measure OD^600 nm^ [25] using a GENios spectrophotometer/fluorometer. Immunohistochemical evaluations were also performed to examine the deposition of type-II, -I, and -X collagen using specific primary antibodies, biotinylated secondary antibodies, and the ABC method with DAB as a chromogen for monitoring under light microscopy (Olympus BX45) [25,44,45]. Control conditions lacking primary antibodies were also evaluated to check for secondary immunoglobulins.

### 2.11. Histomorphometric Analysis

The intensities of X-Gal staining and the percentages of SOX9^+^, TGF-β^+^, and type-II^+^/-I^+^/-X^+^ collagen cells (SOX9-, TGF-β-, and type-II/-I/-X collagen-stained cells to the total cell numbers) were measured at three random sites standardized for their surface using the SIS analySIS program (Olympus) and Adobe Photoshop (Adobe Systems, Unterschleissheim, Germany) [25,45].

### 2.12. Statistical Analysis

Data are provided as mean ± standard deviation (SD) of separate experiments. Each condition was performed in triplicate in three independent experiments per patient. Data were obtained by two individuals blinded with respect to the groups. The t-test and the Mann-Whitney rank sum test were used where appropriate. A *P* value of less than 0.05 was considered statistically significant.

## 3. Results

### 3.1. Effective rAAV Association to Carbon Dots and Release

The reporter rAAV-*lacZ* gene vector was first formulated with the various CDs (CD-1 to CD-4) to examine the ability of these nanoparticles to associate with rAAV and release it over time (up to 10 days, the longest time point evaluated) using Cy labeling and fluorescent evaluation of the vectors in the systems and by measuring the rAAV concentrations in the culture medium via AAV titration ELISA.

**Figure 2:**
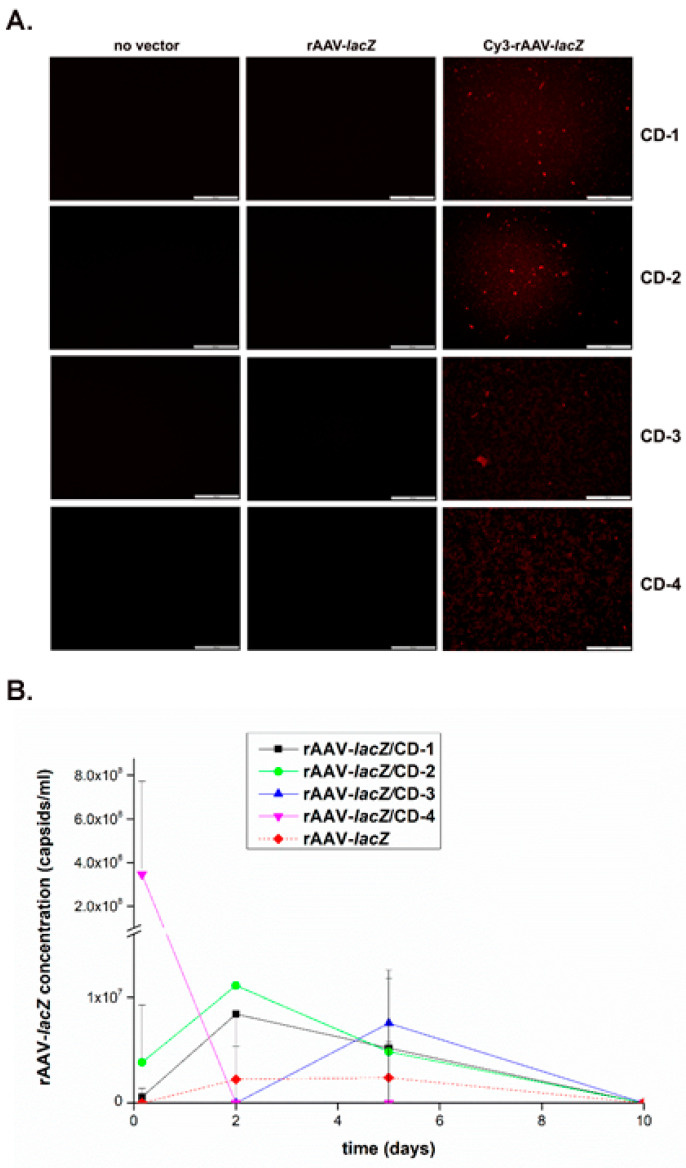
Complexation and release of rAAV vectors from the carbon dots. The rAAV-*lacZ* vector was labeled with Cy3 and formulated with the various CDs (40 μL rAAV, 8 × 10^5^ transgene copies/40 μL CD) and placed in culture over time. (**A**) Cy3-labeled rAAV formulated with the various CDs were examined under live fluorescence after 24 h (magnification ×10; scale bars: 100 μm; all representative data). Control conditions included CD formulations with unlabeled rAAV, CDs lacking rAAV, and absence of CDs. (**B**) rAAV release from the various CDs was monitored by measuring the rAAV concentrations in the culture medium at the denoted time points using an AAV titration ELISA. Free vector treatment was used as a control condition.

Successful formulation of Cy3-labeled rAAV vectors with the different CDs was seen as revealed by the effective detection of live fluorescence in the samples after 24 h relative to the control conditions (CDs formulating unlabeled rAAV and CDs lacking rAAV), without visible difference between CDs or when using Cy3-labeled rAAV vectors in the absence of CD formulation (Figure 2A). Furthermore, all CDs were capable of releasing rAAV over a period of at least 10 days, with CD-2 allowing for the highest early vector release and a good maintenance of vector concentration over time (rAAV-*lacZ*/CD-2) relative to the other CDs (rAAV-*lacZ*/CD-1, rAAV-*lacZ*/CD-3, and rAAV-*lacZ*/CD-4) and versus free vector control (rAAV-*lacZ*) (Figure 2B).

### 3.2. Effective rAAV-Mediated Reporter lacZ Overexpression in hMSCs upon Delivery Assistance by Carbon Dots

The reporter rAAV-*lacZ* gene vector was next formulated with the various CDs to determine the ability of the systems to promote the safe genetic modification of hMSCs over time (up to 10 days, the longest time point evaluated) relative to control conditions (CDs lacking rAAV, i.e., -/CD; free rAAV, i.e., rAAV-*lacZ*; absence of both CDs and rAAV, i.e., -) by monitoring *lacZ* expression using X-Gal staining and via quantitative detection of the β-gal activities in the cells using a Beta-Glo^®^ Assay and by evaluating their viability using the Cell Proliferation Reagent WST-1.

**Figure 3:**
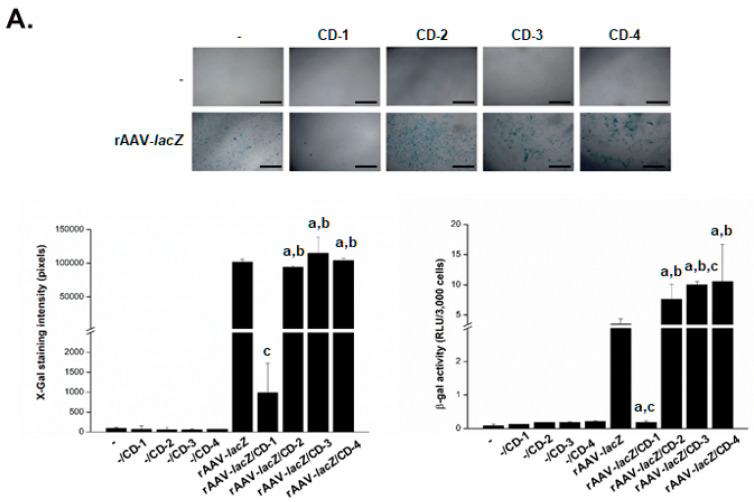

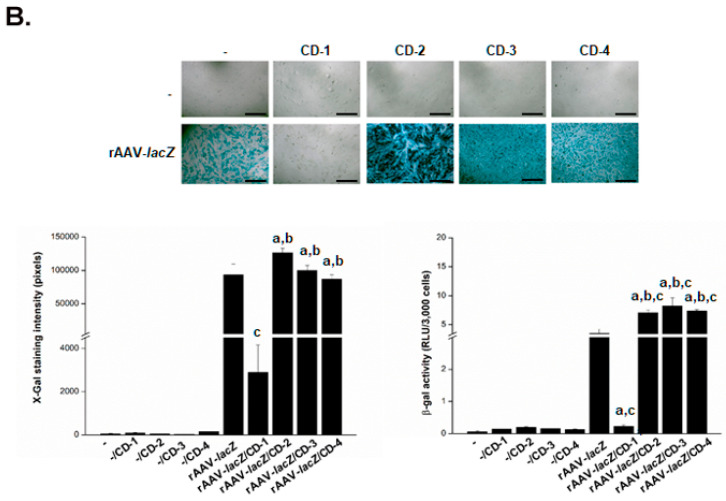
Detection of reporter (*lacZ*) gene overexpression in hMSCs transduced with the rAAV/CD systems. The rAAV-*lacZ* vector (20 μL, 4 × 10^5^ transgene copies) was formulated with the various CDs (CD-1 to CD-4; 20 μL), and the resulting rAAV/CD systems (40 μL, i.e., 4 × 10^5^ transgene copies) were incubated with hMSCs (3000 cells in 96-well plates; MOI = 133) for up to 10 days. Expression of *lacZ* was examined using X-Gal staining (top panel: magnification ×4; scale bars: 500 μm; all representative data) with corresponding histomorphometric analyses (bottom left panel) and using quantitative estimation of the β-gal activities using the Beta-Glo^®^ Assay System (bottom right panel) after one (**A**) and 10 days (**B**). Control conditions included CDs lacking rAAV (-/CD), free rAAV (rAAV-*lacZ*), and absence of both CD and rAAV (-). Statistically significant relative to ^a^ -, ^b^ -/CD and ^c^ rAAV-*lacZ*.

A preliminary, histomorphometric analysis of the early X-Gal staining intensities in the cells on day 1 revealed that the CD-2, CD-3, and CD-4 formulations of rAAV-*lacZ* were capable of promoting *lacZ* expression in the hMSCs without significant difference relative to free vector administration (*P* ≥ 0.050) (Figure 3A). The staining intensities in the cells treated with rAAV-*lacZ*/CD-2, rAAV-*lacZ*/CD-3, and rAAV-*lacZ*/CD-4 increased on day 10, especially when using CD-2 (1.4-fold increase versus day 1; *P* = 0.060), again without difference compared with free rAAV-*lacZ* treatment (*P* ≥ 0.050) (Figure 3B). In marked contrast, delivery of rAAV-*lacZ* in the cells via CD-1 promoted a significant reduction of the staining intensities in hMSCs relative to free vector administration (102.9- and 32.5-fold decrease on days 1 and 10, respectively; always *P* ≤ 0.040) (Figure 3A,B). Overall, these results were supported by a comprehensive, quantitative estimation of the β-gal activities in the cells using the Beta-Glo^®^ Assay, even showing increased activities when providing rAAV-*lacZ* via CD-2, CD-3, or CD-4 versus free vector treatment (up to 2.9- and 2.3-fold difference on days 1 and 10, respectively; always *P* ≤ 0.050) and with reduced activities when using CD-1 (19- and 15.8-fold difference versus free vector administration; always *P* ≤ 0.020) (Figure 3A,B).

**Figure 4:**
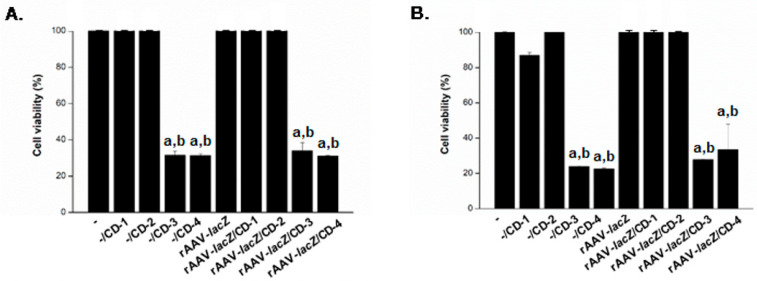
Cell viability in hMSCs transduced with the rAAV/CD systems. The rAAV-*lacZ* vector (20 μL, 4 × 10^5^ transgene copies) was formulated with the various CDs (CD-1 to CD-4; 20 μL) and the resulting rAAV/CD systems (40 μL, i.e., 4 × 10^5^ transgene copies) were incubated with hMSCs (3,000 cells in 96-well plates; MOI = 133) for up to 10 days. Cell viability was examined after one (**A**) and 10 days (**B**) using the Cell Proliferation Reagent WST-1. Control conditions included CDs lacking rAAV (-/CD), free rAAV (rAAV-*lacZ*), and absence of both CD and rAAV (-). Statistically significant relative to ^a^ - and ^b^ rAAV-*lacZ*.

CD-guided delivery of rAAV-*lacZ* to hMSCs using either CD-1 or CD-2 was safe, as revealed by the results of a WST-1 assay, with 100% cell viability preserved on day 1, without significant difference relative to the corresponding control conditions (-, -/CD-1, -CD-2, and free vector administration; always *P* ≥ 0.180) (Figure 4A). In contrast, CD-3 and CD-4 had significantly detrimental effects on cell viability (<32%; always *P* ≤ 0.010 versus all other conditions). Similar observations were noted on day 10, with 100% viability using CD-1 and CD-2, as noted in the corresponding control conditions (always *P* ≥ 0.050), and about 25‒30% viability using CD-3 or CD-4 (always *P* ≤ 0.040 versus all other conditions) (Figure 4B).

### 3.3. Effective rAAV-Mediated SOX9 and TGF-β Overexpression in hMSCs upon Vector Delivery via Carbon Dots

In light of the efficacy and safety of CD-2, the therapeutic rAAV-FLAG-h*sox9* and rAAV-hTGF-β were next formulated independently with these nanoparticles (rAAV-FLAG-h*sox9*/CD-2 and rAAV-hTGF-β/CD-2, respectively) to determine the ability of the system to promote the overexpression of each candidate gene (SOX9, TGF-β) in hMSCs over time (up to 21 days, the longest time point evaluated) relative to control conditions (CD-2 lacking rAAV, i.e., -/CD-2, CD-2 formulating rAAV-*lacZ*, i.e., rAAV-*lacZ*/CD-2) using immunocytochemical detection of each transgene product. Therapeutic (SOX9, TGF-β) rAAV vectors without CD-2 were not included, as controls because they have been characterized in similar culture conditions in earlier studies [44,45], and in light of the quantitative estimation of the β-gal activities in the cells on day 10, significantly increased activities with rAAV-*lacZ*/CD-2 versus free rAAV-*lacZ* vector treatment were revealed (Figure 3B).

**Figure 5:**
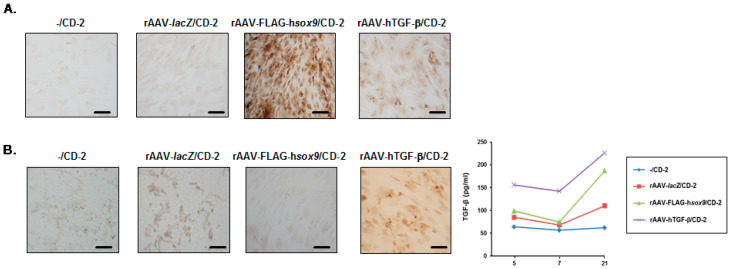
Detection of therapeutic (SOX9, TGF-β) gene overexpression in hMSCs transduced with the rAAV/CD-2 system. The rAAV-FLAG-h*sox9*, rAAV-hTGF-β, and rAAV-*lacZ* vectors (40 μL each vector, 8 × 10^5^ transgene copies) were formulated with CD-2 (40 μL), and the resulting rAAV/CD systems (80 μL, i.e., 8 × 10^5^ transgene copies) were incubated with hMSCs (10,000 cells in 48-well plates; MOI = 80) for up to 21 days. SOX9 (**A**) and TGF-β (**B**) expression was examined using immunocytochemistry (A, B; magnification ×20; scale bars: 50 μm; all representative data) and using specific (TGF-β) ELISA (B). rAAV-*lacZ*/CD-2 and CD-2 lacking rAAV were used as controls.

An immunocytochemical analysis of SOX9 expression in the cells revealed that administration of rAAV-FLAG-h*sox9* to hMSCs via CD-2 led to significantly higher levels of SOX9 expression relative to all other conditions after 21 days (65-, 43.3-, and 1.8-fold difference using rAAV-FLAG-h*sox9*/CD-2 versus -/CD-2, rAAV-*lacZ*/CD-2, and rAAV-hTGF-β/CD-2, respectively; always *P* ≤ 0.001) (Figure 5A and Table 2). An evaluation of TGF-β expression using immunocytochemistry also showed that delivery of rAAV-hTGF-β to hMSCs via CD-2 led to significantly higher levels of TGF-β expression relative to all other conditions after 21 days (10.3-, 6.8-, and 9.4-fold difference using rAAV-hTGF-β/CD-2 versus -/CD-2, rAAV-*lacZ*/CD-2, and rAAV-FLAG-h*sox9*/CD-2, respectively; always *P* ≤ 0.001) (Figure 5B and Table 2). This result was corroborated by an estimation of the levels of TGF-β production in the cells using ELISA, with up to 2.8-, 2.8-, and 3.8-fold higher TGF-β secretion levels when using rAAV-hTGF-β/CD-2 after 5, 7, and 21 days, respectively, versus all other conditions (always *P* ≤ 0.001) (Figure 5B).

### 3.4. Effects of rAAV-Mediated SOX9 and TGF-β Overexpression on the Biological Activities in hMSCs upon Vector Delivery via Carbon Dots

The ability of the delivery systems to trigger the biological activities (cell proliferation, matrix deposition) in hMSCs over time (21 days) relative to control conditions (-/CD-2, rAAV-*lacZ*/CD-2) was then examined with the two formulations rAAV-FLAG-h*sox9*/CD-2 and rAAV-hTGF-β/CD-2 by evaluating cell viability using the Cell Proliferation Reagent WST-1 and matrix deposition via spectrophotometric detection of alcian blue staining (glycosaminoglycans) and via immunocytochemical detection of type-II, -I, and -X collagen expression.

**Figure 6:**
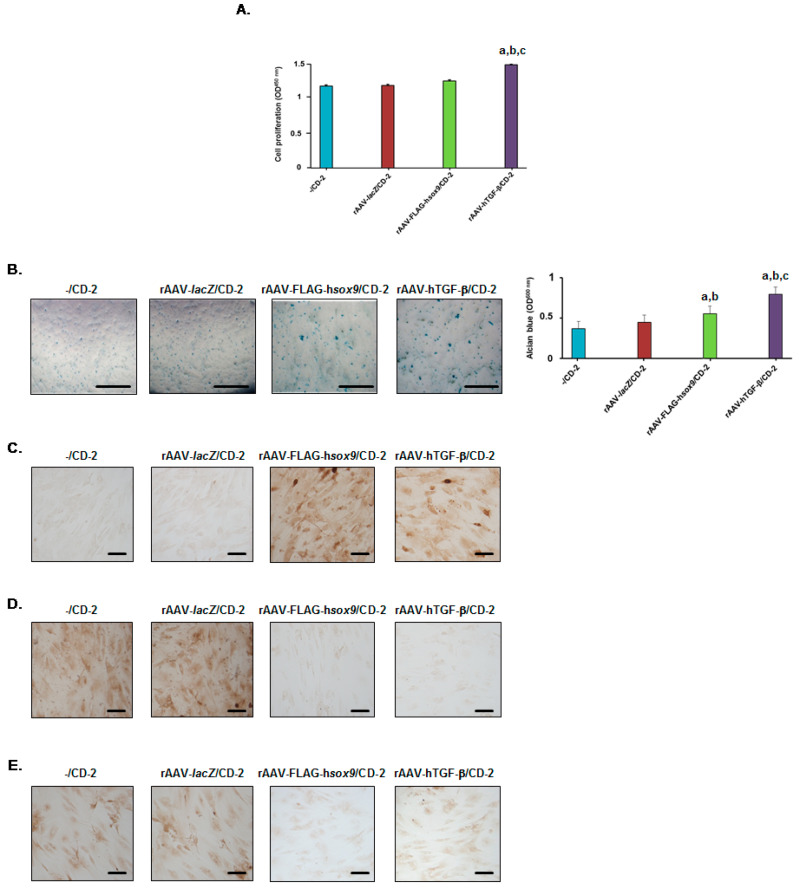
Biological activities in hMSCs transduced with the rAAV/CD-2 system. The rAAV-FLAG-h*sox9*, rAAV-hTGF-β, and rAAV-*lacZ* vectors (40 μL each vector, i.e., 8 × 10^5^ transgene copies) were formulated with CD-2 (40 μL), and the resulting rAAV/CD systems (80 μL) were incubated with hMSCs (10,000 cells in 48-well plates; MOI = 80) for up to 21 days. Cell proliferation was examined using the Cell Proliferation Reagent WST-1 (**A**), glycosaminoglycans using alcian blue staining (light microscopy; magnification ×4; scale bars: 200 μm; all representative data) with spectrophotometric analysis after solubilization (histograms) (**B**), and the deposition of type-II collagen (**C**), type-I collagen (**D**), and type-X collagen (**E**) using immunocytochemistry (magnification ×20; scale bars: 50 μm; all representative data). rAAV-*lacZ*/CD-2 and CD-2 lacking rAAV were used as controls. Statistically significant relative to ^a^ -/CD-2, ^b^ rAAV-*lacZ*/CD-2, and ^c^ rAAV-FLAG-h*sox9*.

Administration of rAAV-hTGF-β in hMSCs via CD-2 led to significantly higher levels of cell proliferation relative to all other conditions after 21 days (1.3-, 1.3-, and 1.2-fold difference using rAAV-hTGF-β/CD-2 versus -/CD-2, rAAV-*lacZ*/CD-2, and rAAV-FLAG-h*sox9*/CD-2, respectively; always *P* ≤ 0.001), while no difference was seen with rAAV-FLAG-h*sox9*/CD-2 (*P* ≥ 0.065 versus -/CD-2 or rAAV-*lacZ*/CD-2) (Figure 6A). Delivery of either rAAV-FLAG-h*sox9* or rAAV-hTGF-β in hMSCs via CD-2 led to significantly higher levels of glycosaminoglycans relative to all other conditions after 21 days (1.3- and 1.2-fold difference using rAAV-FLAG-h*sox9*/CD-2 versus -/CD-2 and rAAV-*lacZ*/CD-2, respectively, always *P* ≤ 0.002; 1.8- and 1.7-fold difference using rAAV-hTGF-β/CD-2 versus -/CD-2 and rAAV-*lacZ*/CD-2, respectively, always *P* ≤ 0.001), with a stronger effect of TGF-β relative to SOX9 (1.4-fold difference; *P* ≤ 0.001) (Figure 6B). Delivery of either rAAV-FLAG-h*sox9* or rAAV-hTGF-β to hMSCs via CD-2 led to significantly higher levels of type-II collagen expression relative to all other conditions after 21 days (17.8- and 15.4-fold difference using rAAV-FLAG-h*sox9*/CD-2 versus -/CD-2 and rAAV-*lacZ*/CD-2, respectively, always *P* ≤ 0.001; 14.4- and 12.5-fold difference using rAAV-hTGF-β/CD-2 versus -/CD-2 and rAAV-*lacZ*/CD-2, respectively, always *P* ≤ 0.001), with a stronger effect of SOX9 relative to TGF-β (1.2-fold difference; *P* ≤ 0.002) (Figure 6C and Table 2).

Interestingly, administration of rAAV-FLAG-h*sox9* or of rAAV-hTGF-β in hMSCs via CD-2 led to significantly lower levels of type-I collagen expression relative to all other conditions after 21 days (20.1- and 20.2-fold difference using rAAV-FLAG-h*sox9*/CD-2 versus -/CD-2 and rAAV-*lacZ*/CD-2, respectively, always *P* ≤ 0.001; 22.7- and 22.9-fold difference using rAAV-hTGF-β/CD-2 versus -/CD-2 and rAAV-*lacZ*/CD-2, respectively, always *P* ≤ 0.001), without difference between SOX9 and TGF-β (*P* = 0.319) (Figure 6D and Table 2). Similar results were noted when analyzing type-X collagen expression (6.2- and 6.1-fold difference using rAAV-FLAG-h*sox9*/CD-2 versus -/CD-2 and rAAV-*lacZ*/CD-2, respectively, always *P* ≤ 0.001; 5.7-fold difference using rAAV-hTGF-β/CD-2 versus -/CD-2 or rAAV-*lacZ*/CD-2, respectively, always *P* ≤ 0.001), again without difference between SOX9 and TGF-β (*P* = 0.257) (Figure 6E and Table 2).

## 4. Discussion

Biomaterial-guided gene delivery using clinically adapted rAAV vectors [10,11,24,25,26,27,28,29,30,31,32,33] is an emerging, potent approach to treat focal cartilage lesions by non-invasive transfer and overexpression of chondroregenerative factors. In the present study, we examined the feasibility of providing independent rAAV constructs coding for the highly chondroreparative SOX9 transcription factor [43] and TGF-β [5,6] to hMSCs via carbon dots (CDs) as a means to stimulate the biological activities in these cells, an advantageous source of progenitor cells to enhance the intrinsic healing processes in sites of cartilage damage [5,6,7].

The present findings show, for the first time to our best knowledge, that CDs may be effective systems to successfully formulate and release rAAV gene transfer vectors. Among all the CDs tested here, CD-2, a carbonaceous nanoparticle prepared using pyrolysis at normal pressure of a mixture of CA, mPEG_550_, and DMEDA, allowed for the highest intracellular vector release with a good over time maintenance for at least 10 days, the longest time point examined. Equally important, CD-2 was able to promote the effective and sustained modification of hMSCs when used to deliver a reporter (rAAV-*lacZ*) gene vector for at least 10 days (up to 2.2-fold increase in *lacZ* expression relative to free vector treatment) in a safe manner (100% cell viability, presumably due to the presence of the PEG protective shield around the particles), reaching levels similar to those noted with other nano-sized systems for rAAV delivery in hMSCs [24]. In contrast, the genetic modification of hMSCs using CD-3 or CD-4 was associated with decreased levels of cell viability, while CD-1 led to a reduction of gene transfer efficiency relative to free vector administration and other control conditions.

The results next demonstrate that the optimal CD-2 nanoparticles were further capable of promoting the delivery of rAAV vectors coding for the therapeutic *sox9* and TGF-β candidate genes in hMSCs, promoting a significant overexpression of each transgene in the cells over an extended period of time (about 97.5% SOX9^+^ cells using rAAV-FLAG-h*sox9*/CD-2 and 79.8% TGF-β^+^ cells with rAAV-hTGF-β/CD-2 after 21 days) relative to control treatments (≤7.8% and ≤11.8% transgene-expressing cells in the -/CD-2 and rAAV-*lacZ*/CD-2 conditions, respectively), higher than upon free rAAV *sox9* application (80–85%) [44] and comparable to free TGF-β gene transfer (80%) [45]. Yet, the levels of TGF-β produced via rAAV-hTGF-β/CD-2 (155–225 pg/mL) were 4- to 56-fold higher than those achieved upon free rAAV TGF-β gene transfer (17–24 pg/mL) [45]. This result is probably due to the difference of vector doses applied (MOI = 80–133 here compared with MOI = 4–20 using free vector gene administration, i.e., a 4- to 33-fold difference), but it reflects the improvement of TGF-β production via CD-2-guided rAAV gene transfer, as application of the current vector dose in a free form would have only raised 70‒160 pg/mL of growth factor in cells versus 155–225 pg/mL here via CD-2 (i.e., a 1.4- to 2.2-fold difference). Interestingly, application of rAAV-hTGF-β/CD-2 resulted in the detection of 52.8% SOX9^+^ cells, probably due to an upregulation of SOX9 expression in response to TGF-β production via rAAV/CD-2, as previously noted when using TGF-β in its recombinant form (rTGF-β) [48] or upon free rAAV TGF-β gene transfer [45], while no effects of SOX9 overexpression were seen on the levels of TGF-β. Effective SOX9 and TGF-β overexpression via CD-2-guided gene delivery led to increased levels of cartilage matrix production in the cells (glycosaminoglycan and type-II collagen expression) over time (21 days) relative to the control conditions, concordant with the respective pro-anabolic activities of SOX9 [43] and TGF-β [5,6,7], with observations showing short-term effects only of nonviral SOX9 gene transfer using arginine-based CDs (14 days) [42], and with our previous findings using free rAAV *sox9* or TGF-β gene transfer [44,45]. Furthermore, application of rAAV-hTGF-β/CD-2 had a significant influence on hMSC proliferation, in good agreement with the properties of the growth factor [6] and with our previous observations using free rAAV TGF-β gene transfer [45]. In contrast, rAAV-FLAG-h*sox9*/CD-2 had no impact on such a process, consistent with the activities of SOX9 [49] and with our findings via free rAAV *sox9* gene transfer [44]. Interestingly, CD-2-guided delivery of either rAAV-FLAG-h*sox9* or rAAV-hTGF-β advantageously prevented the deposition of type-I and -X collagen in hMSCs over time versus control treatments, concordant with the effects of SOX9 [49] and with results obtained using free rAAV *sox9* gene transfer [44], but in contrast to findings using rTGF-β [5] or upon free rAAV TGF-β gene delivery [45]. This might be due to differences of culture conditions and cell environment (monolayer hMSC cultures here versus three-dimensional hMSC cultures in free rAAV TGF-β gene transfer setting) [45] or to the differences between the levels of TGF-β achieved here via rAAV-hTGF-β/CD-2 (155–225 pg/mL) and the amounts of rTGF-β applied elsewhere (10 ng/mL, i.e., a 44- to 65-fold difference) [5].

In conclusion, the present work reports the possibility of transferring therapeutic rAAV (SOX9 or TGF-β) gene vectors to reparative hMSCs using optimal carbon-based nanoparticles as a novel, off-the-shelf system for cartilage repair. It will be interesting to extend the current approach in the future using adipose-derived hMSCs as these cells can be harvested at a 1000-fold higher yield in a less invasive manner than bone marrow-derived MSCs while displaying longer life-span and higher proliferative capacity and carrying micro-RNAs that regulate tissue inflammation and cell interplays [50,51]. Analyses are ongoing to test the value of the approach in a three-dimensional environment (high-density cultures) using single and combined CD-2-assisted rAAV SOX9/TGF-β gene transfer to potentiate the effects of the two factors on cell proliferation (TGF-β) and matrix deposition (glycosaminoglycans with TGF-β superiority and type-II collagen with SOX9 superiority) [52] and next in an orthotopic in vivo model of cartilage defect [14,16,53,54]. Such evaluations will provide insights into the potential benefits of CDs over other scaffolds (collagen, hyaluronic acid) or treatments like autologous platelet-rich plasma [50,55,56] for translational cartilage regeneration. Overall, this evaluation provides original evidence on the ability of CD-guided therapeutic rAAV gene transfer in regenerative hMSCs as platforms for therapy of cartilage defects in translational protocols.

## Figures and Tables

**Table 1:** Characteristics of the various carbon dots (CD-1 to CD-4) employed in the study.

Name	Starting Material (w/w)	Activation Mode	Size (nm) ^a^		Potential ^a^ (mV)
			DLS	TEM	
CD-1	CA/PEHA (1/4)	(1) 30 min at 180 °C ^b^ (2) 30 min at 230 °C ^b^	36.4 ± 12.0	17.9	+18.6 ± 0.9
CD-2	CA/mPEG_550_/DMEDA (1/3/3)	(1) 30 min at 180 °C ^b^ (2) 30 min at 230 °C ^b^	17.7 ± 0.9	16.3	+26.9 ± 1.6
CD-3	CA/bPEI_600_/mPEG_2000_ (1/4/1)	MW 620 W, 190 s ^c^	13.3 ± 0.4	-	+29.4 ± 0.4
CD-4	CA/bPEI_600_ (1/4)	MW 620 W, 120 s ^c^	11.7 ± 0.9	-	+37.6 ± 3.2

^a^ Measured at 1.0 mg/mL in 1.5 mM NaCl, pH 7.4. ^b^ Reactions were conducted under conventional heating. ^c^ Reactions were conducted in a domestic microwave oven.

**Table 2:** Histomorphometric analyses in hMSCs transduced with the rAAV/CD-2 systems.

Parameter	-/CD-2	rAAV-*lacZ*/CD-2	rAAV-FLAG-h*sox9*/CD-2	rAAV-hTGF-/CD-2
SOX9	1.5 ± 0.6	2.3 ± 0.5	97.5 ± 1.3 ^a,b^	52.8 ± 2.2 ^a,b,c^
TGF-	7.8 ± 3.1	11.8 ± 2.4	8.5 ± 1.3	79.8 ± 3.9 ^a,b,c^
Type-II collagen	4.8 ± 2.5	5.5 ± 2.6	84.8 ± 2.2 ^a,b^	68.5 ± 4.5 ^a,b,c^
Type-I collagen	85.3 ± 2.2	85.8 ± 2.6	4.3 ± 1.7 ^a,b^	3.8 ± 1.0 ^a,b^
Type-X collagen	73.3 ± 1.7	72.3 ± 1.7	11.8 ± 1.7 ^a,b^	12.8 ± 1.7 ^a,b^

Values are given as mean ± SD. All parameters are in % of positively (SOX9^+^, TGF-β^+^, type-II^+^/-I^+^/-X^+^ collagen) stained cells to the total cell numbers. Statistically significant relative to ^a^ -/CD-2, ^b^ rAAV-*lacZ*/CD-2 and ^c^ rAAV-FLAG-h*sox9*.
